# Successful Salvage of a Mutilating Hand Injury With Multiple Digital Crush and Open Fractures: A Case Report

**DOI:** 10.7759/cureus.106778

**Published:** 2026-04-10

**Authors:** Nay Aung Zin, Zin Myo Tun, Phyu S Thwin, Khadheeja Abdulla, Tun Aung Khine, Nang Hsu Yi Htwe

**Affiliations:** 1 Orthopaedics and Traumatology, Kulhudhuffushi Regional Hospital, Kulhudhuffushi, MDV; 2 Orthopaedics and Traumatology, Pun Hlaing International Hospital, Yangon, MMR; 3 Emergency Medicine, Kulhudhuffushi Regional Hospital, Kulhudhuffushi, MDV; 4 General Surgery, Kulhudhuffushi Regional Hospital, Kulhudhuffushi, MDV; 5 Emergency Medicine, Indira Gandhi Memorial Hospital, Malé, MDV; 6 General Practice, Damaw Thada Social Clinic, Yangon, MMR

**Keywords:** crush injury, digital amputation, functional outcome, hand reconstruction, hand trauma, kirschner wire fixation, mutilating hand injury, orthopedic surgery

## Abstract

Mutilating hand injuries involving multiple digits present significant reconstructive challenges, particularly in the setting of severe crush mechanisms and vascular compromise. We report the case of an 18-year-old right-hand dominant male manual laborer who sustained a devastating crush injury to his right hand after entrapment under a concrete block. The injury involved near-amputations of the index, middle, and ring fingers, with complete disruption of digital neurovascular bundles and associated open fractures.

Given the non-viability of distal segments and extensive soft tissue damage, replantation was not feasible. The patient underwent aggressive debridement, skeletal stabilization with Kirschner wires, and revision amputation with soft tissue reconstruction. Early rehabilitation was initiated postoperatively.

Despite significant digital shortening, the patient achieved satisfactory functional recovery, including the ability to form a functional grip. This case highlights the importance of prioritizing functional outcomes over anatomical preservation in severe mutilating hand injuries and demonstrates that acceptable results can be achieved with appropriate surgical decision-making and rehabilitation.

## Introduction

Mutilating hand injuries are complex and often involve a combination of bone, tendon, vascular, and soft tissue damage. These injuries are frequently associated with high-energy mechanisms such as crush or avulsion trauma, resulting in extensive tissue destruction and compromised viability.

While replantation remains the gold standard for certain hand injuries, particularly in cases involving the thumb or multiple digits, it is not always feasible in the presence of severe crush injury and segmental tissue loss. In such cases, the goal shifts toward achieving a functional hand through appropriate debridement, stabilization, and reconstruction. We present a case of severe mutilating hand injury involving multiple digits managed successfully with thorough debridement and skeletal stabilization, emphasizing functional salvage and rehabilitation.

## Case presentation

An 18-year-old right-hand dominant male manual laborer presented to the emergency department following a crush injury to his right hand sustained when it was trapped under a heavy concrete block. On examination, there was a severe mutilating injury involving the index, middle, and ring fingers. The index finger demonstrated near-complete amputation at the distal interphalangeal (DIP) joint level with extensive crush injury, rendering identification of neurovascular structures impossible. The middle and ring fingers were also nearly amputated, with only a portion of the flexor tendon and dorsal skin attachments remaining. Both digital neurovascular bundles in these digits were completely transected.

**Figure 1:**
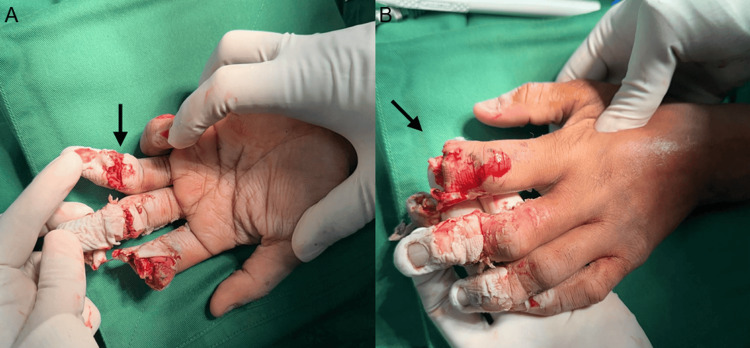
Intraoperative findings (A) Palmar view demonstrating severe crush injury with near-amputation of multiple digits. The arrow indicates the ring finger, showing extensive soft tissue loss, devitalized tissue, and disruption of normal anatomical structures consistent with a non-viable distal segment. (B) Dorsal-oblique view of the injured hand. The arrow highlights the index finger, demonstrating near-complete amputation at the distal level with exposed soft tissue and gross contamination following crush injury.

There were no associated nail bed injuries, but open fractures involving the proximal phalanges of the middle and ring fingers. The distal segments of all affected digits were non-perfused, with no evidence of capillary refill, consistent with complete disruption of arterial inflow. Sensory examination was not possible due to the extent of injury (Figure 1).

Radiographic evaluation confirmed open fractures of the proximal phalanges of the middle and ring fingers, with associated soft tissue loss. Given the extent of soft tissue destruction, vascular compromise, and non-viability of distal segments, the injury was classified as a severe mutilating hand injury with multiple digital crush and near-amputation components.

Management and surgical procedure

**Figure 2:**
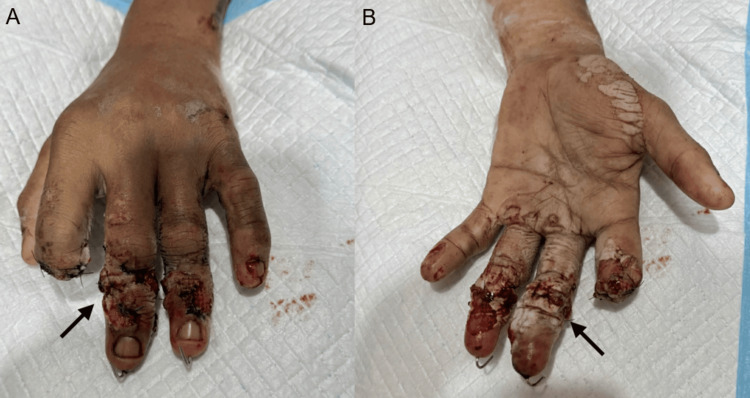
Immediate postoperative appearance (A) Dorsal view of the right hand immediately after surgical management, demonstrating sutured wounds over the middle and ring fingers following debridement and skeletal stabilization. The arrow indicates the middle finger, showing soft tissue reconstruction with Kirschner wire fixation and primary closure. (B) Palmar view illustrating the postoperative condition of the digits. The arrow highlights the middle finger showing circumferential sutured wounds with preserved soft tissue coverage following debridement and fixation. The index finger demonstrates revision amputation at the distal level with a well-contoured stump.

Upon presentation, the patient was managed according to advanced trauma principles. The injured hand was irrigated, covered with sterile dressings, and immobilized. Intravenous broad-spectrum antibiotics and tetanus prophylaxis were administered promptly. The patient presented approximately five hours after injury and underwent emergency surgical intervention shortly thereafter (Figure 2).

Under regional anesthesia with tourniquet control, thorough exploration of the injured digits was performed. Extensive crush injury with devitalized soft tissue, contamination, and complete disruption of the digital arteries and nerves of the index, middle, and ring fingers were confirmed. The distal segments of all affected digits were non-viable, with no evidence of perfusion.

Aggressive and meticulous debridement was carried out, excising all non-viable tissues while preserving as much viable skin and soft tissue as possible. Given the severe crush component, segmental loss of neurovascular structures, and absence of suitable vessels for microvascular repair, replantation or revascularization was deemed not feasible.

**Figure 3:**
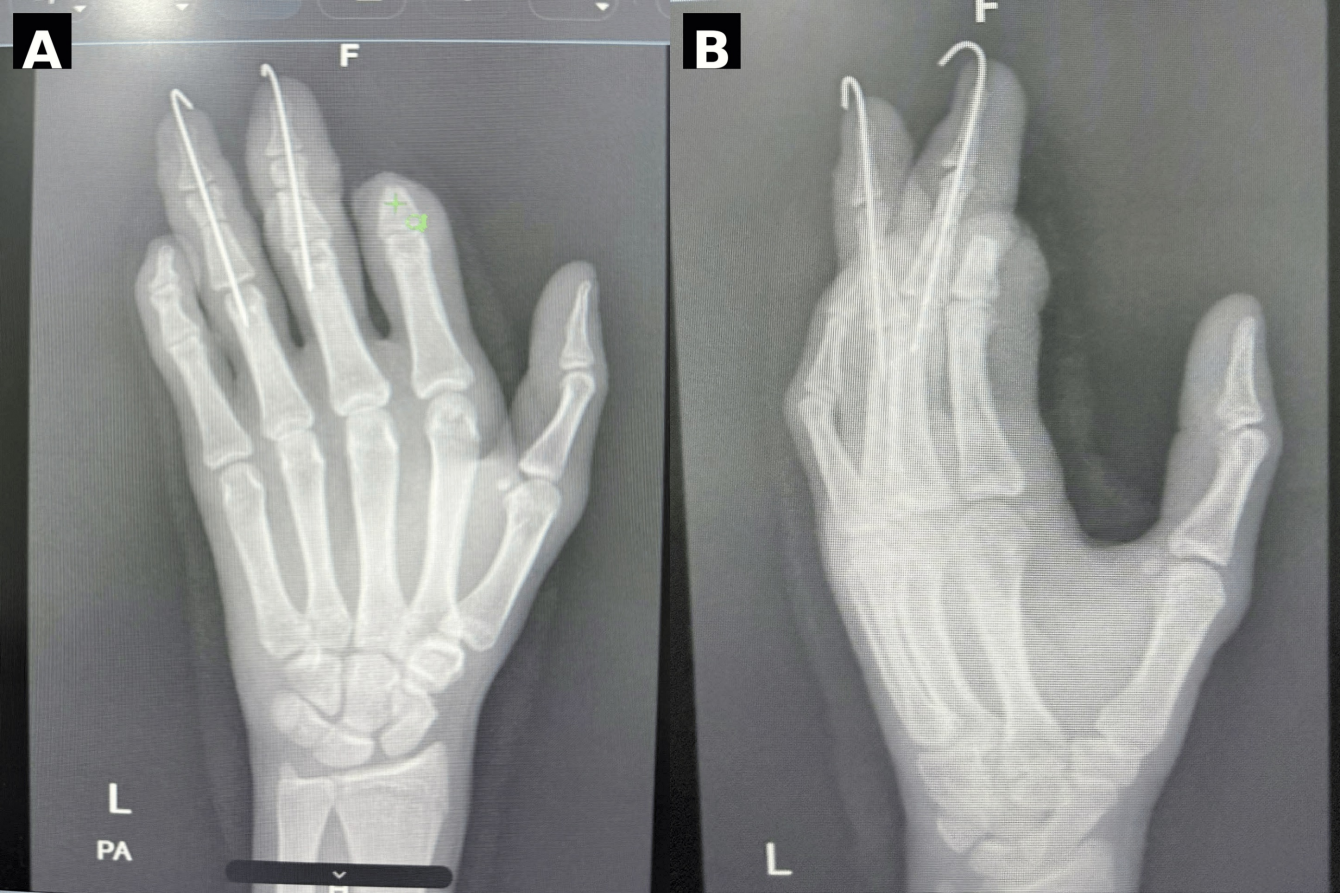
Postoperative radiographic evaluation (A) Anteroposterior (PA) view of the left hand demonstrating skeletal stabilization of the middle and ring fingers with Kirschner wires (K-wires). Length and alignment of the remaining phalanges are maintained. (B) Oblique view confirming satisfactory positioning of the K-wires and overall alignment of the digits after fixation.

Skeletal stabilization was achieved for the middle and ring fingers using Kirschner wires (K-wires 1.2 mm inserted using the antegrade technique) following appropriate shortening to healthy bone ends to facilitate soft tissue coverage and reduce tension. The index finger, which was severely crushed at the DIP level with non-reconstructable tissue, underwent revision amputation with appropriate contouring of the stump. The K-wires were maintained for four weeks to allow adequate fracture healing (Figure 3).

Soft tissue management focused on achieving durable coverage. Primary closure was performed where feasible after adequate shortening, while preserving maximal length and function. The nail bed injuries were addressed, and non-salvageable nail bed tissue was excised. Hemostasis was secured, and sterile dressings were applied.

Postoperatively, the hand was immobilized in a functional position. Postoperatively, the patient was administered intravenous broad-spectrum antibiotics for three days, followed by oral antibiotics for five days, considering the open fractures and high risk of contamination. K-wire removal was performed at four weeks postoperatively. Early involvement of hand therapy was initiated once soft tissue healing permitted, focusing on edema control, range of motion, and functional rehabilitation.

**Figure 4:**
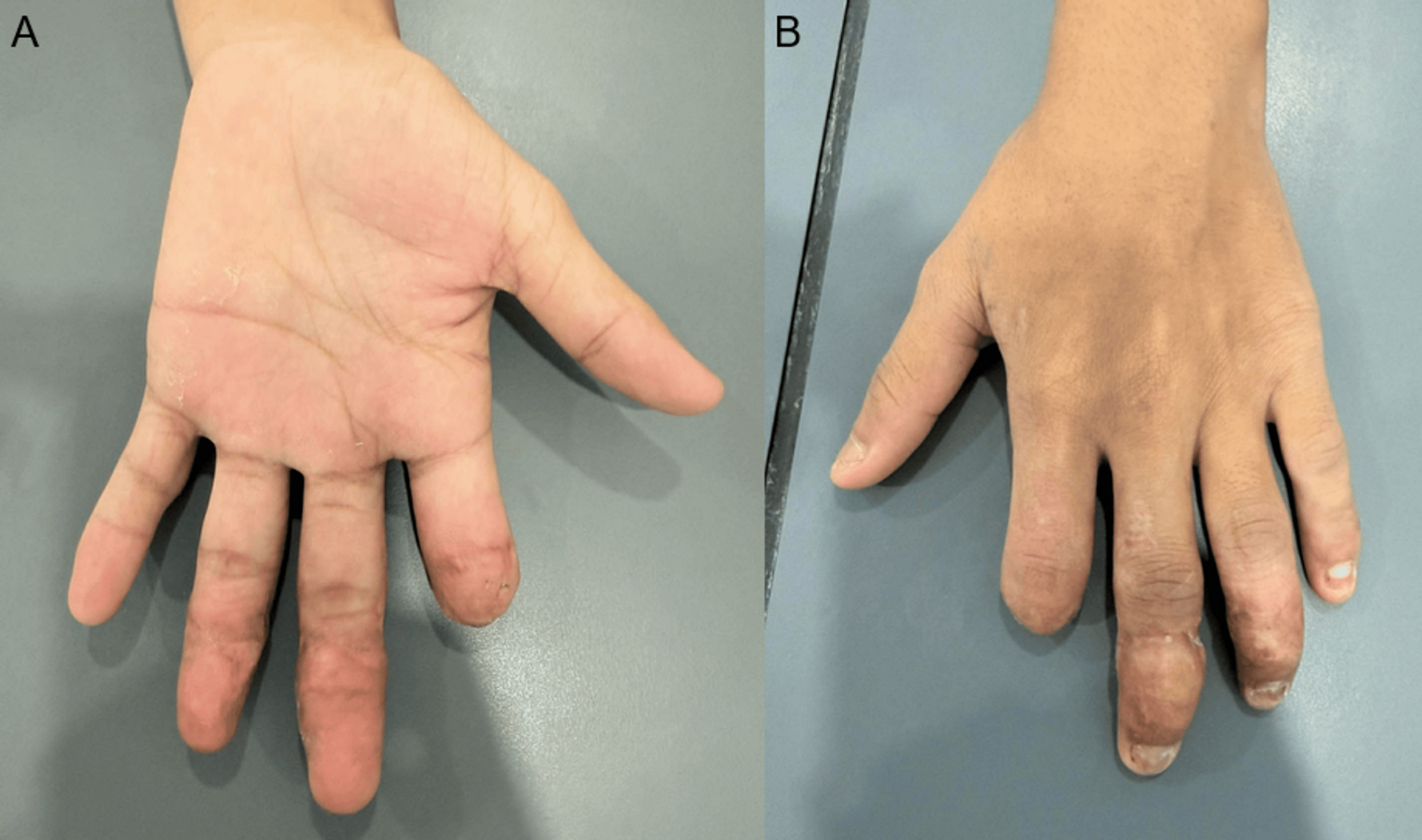
Final clinical outcome at 6 months (A) Palmar view of the right hand at final follow-up demonstrating healed soft tissues and stable digital stumps following surgical management. Despite shortening of the index finger, adequate finger positioning is maintained. (B) Dorsal view showing satisfactory alignment of the digits with well-healed scars and preserved hand contour.

**Figure 5:**
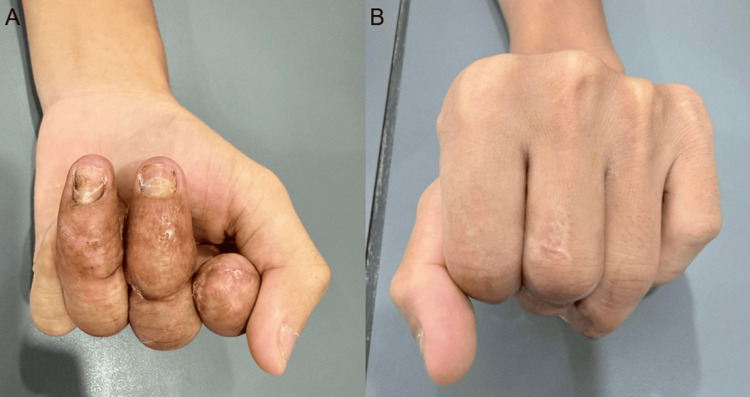
Functional outcome at final follow-up (A) Flexion (grip) view of the right hand demonstrating the ability to achieve a functional grasp despite significant shortening of the index finger. The digits are well healed with adequate soft tissue coverage. (B) Dorsal view in flexion showing maintained alignment of the digits and preservation of overall hand function, with healed scars and satisfactory contour.

At the six-month follow-up, the patient demonstrated satisfactory wound healing and was able to achieve a functional grip despite digital shortening, with an acceptable cosmetic outcome (Figures 4, 5).

## Discussion

Mutilating injuries of the hand involving multiple digits remain among the most challenging conditions in reconstructive surgery. The primary objective in such cases is to restore meaningful hand function that allows the patient to return to daily activities and work [1].

Replantation and revascularization are well-established techniques in hand surgery; however, their success is significantly influenced by the mechanism of injury. Crush injuries, as seen in this case, are associated with extensive soft tissue damage, vascular disruption, and poorer outcomes compared to sharp injuries [2]. In our patient, complete disruption of the digital arteries and nerves with the absence of distal perfusion indicated a particularly severe injury pattern.

Despite these unfavorable factors, selected patients-especially young individuals with high functional demands-may still benefit from an aggressive salvage approach. Previous studies have emphasized that even limited functional recovery can substantially improve quality of life and independence [3].

Management of such injuries requires a systematic approach, including meticulous debridement, skeletal stabilization, and soft tissue reconstruction where feasible [4]. Preservation of viable tissue while removing all devitalized structures is essential to optimize healing and prevent complications.

K-wire fixation remains a reliable method for stabilizing phalangeal fractures, providing adequate alignment with minimal additional trauma to soft tissues [5]. This technique was effectively utilized in our case following bone shortening.

Early and structured rehabilitation played a crucial role in functional recovery. A supervised physiotherapy program focusing on range of motion, edema control, and progressive strengthening was initiated after adequate wound healing, contributing significantly to the final outcome. Early rehabilitation has been shown to improve range of motion and overall functional outcomes following complex hand injuries [6]. In this case, the patient demonstrated steady improvement with good adherence to the prescribed rehabilitation protocol.

Outcomes following severe hand trauma depend on multiple factors, including injury severity, ischemia time, and surgical expertise [7]. Large clinical series have reported variable success rates in digital replantation, particularly in crush injuries where vascular damage is extensive [8].

Meta-analyses have further confirmed that crush mechanisms are associated with lower survival rates compared to clean-cut injuries [9]. In selected cases, alternative reconstructive strategies, including simplified vascular approaches, may be considered to improve outcomes [10]. This case demonstrates that even in the setting of extensive crush injury with complete neurovascular disruption, a tailored salvage approach can result in a functional and usable hand.

## Conclusions

Severe mutilating hand injuries with multiple digital crush and vascular disruption may not be amenable to replantation. In such cases, prioritizing functional salvage through aggressive debridement, skeletal stabilization, and early rehabilitation can result in satisfactory outcomes. This case highlights the importance of individualized decision-making and the principle of function over form in complex hand injuries.
